# Nanobodies against Pfs230 block *Plasmodium falciparum* transmission

**DOI:** 10.1042/BCJ20220554

**Published:** 2022-12-22

**Authors:** Melanie H. Dietrich, Mikha Gabriela, Kitsanapong Reaksudsan, Matthew W. A. Dixon, Li-Jin Chan, Amy Adair, Stephanie Trickey, Matthew T. O'Neill, Li Lynn Tan, Sash Lopaticki, Julie Healer, Sravya Keremane, Alan F. Cowman, Wai-Hong Tham

**Affiliations:** 1 Infectious Diseases and Immune Defence Division, The Walter and Eliza Hall Institute of Medical Research, Parkville, Victoria 3052, Australia; 2 Department of Medical Biology, The University of Melbourne, Melbourne, Victoria 3010, Australia; 3 Department of Infectious Diseases, Peter Doherty Institute for Infection and Immunity, University of Melbourne, Melbourne, Australia

**Keywords:** 6-cysteine proteins, crystallography, malaria, nanobodies, *Plasmodium falciparum*

## Abstract

Transmission blocking interventions can stop malaria parasite transmission from mosquito to human by inhibiting parasite infection in mosquitos. One of the most advanced candidates for a malaria transmission blocking vaccine is Pfs230. Pfs230 is the largest member of the 6-cysteine protein family with 14 consecutive 6-cysteine domains and is expressed on the surface of gametocytes and gametes. Here, we present the crystal structure of the first two 6-cysteine domains of Pfs230. We identified high affinity Pfs230-specific nanobodies that recognized gametocytes and bind to distinct sites on Pfs230, which were isolated from immunized alpacas. Using two non-overlapping Pfs230 nanobodies, we show that these nanobodies significantly blocked *P. falciparum* transmission and reduced the formation of exflagellation centers. Crystal structures of the transmission blocking nanobodies with the first 6-cysteine domain of Pfs230 confirm that they bind to different epitopes. In addition, these nanobodies bind to Pfs230 in the absence of the prodomain, in contrast with the binding of known Pfs230 transmission blocking antibodies. These results provide additional structural insight into Pfs230 domains and elucidate a mechanism of action of transmission blocking Pfs230 nanobodies.

## Introduction

Sexual stage development of *Plasmodium falciparum* is essential for the transmission of the malaria parasite from mosquito to human. When a female *Anopheles* mosquito takes a blood meal, gametocytes, which are the sexual forms of the malaria parasites, are taken up into the mosquito midgut. The female gametocyte develops into a macrogamete whilst the male gametocyte undergoes three rounds of DNA replication and mitosis to form eight highly motile microgametes, a process known as exflagellation [1,2]. Exflagellation can be induced *in vitro* and is characterized by the formation of red blood cell clusters around the activated microgamete [3]. This exflagellation center is thought to facilitate microgamete release from the residual body and the subsequent mating process [3]. Fertilization occurs upon fusion of the microgamete and macrogamete, leading to the formation of a zygote. Zygotes elongate to form ookinetes, which traverse through the mosquito midgut epithelial cells and then develop into oocysts on the outer layer of the mosquito midgut. Oocysts rupture and release sporozoites, which travel to the mosquito's salivary glands, ready to infect another human during the next blood meal. Blocking these stages of sexual development can inhibit malaria parasite transmission.

Pfs230 is one of the leading transmission-blocking malaria vaccine candidates in development [4]. Pfs230 is expressed in both male and female gametocytes from stage II gametocytes until the end of fertilization [5]. In gametocytes, Pfs230 is expressed with a N-terminal prodomain that is processed during gametogenesis, prior to the emergence of the gametes from red blood cells in the mosquito midgut [6,7]. Compared with orthologs of other *Plasmodium* species, the prodomain region varies highly in length and sequence containing species-specific oligopeptide repeats [8]. The oligopeptide repeats are immunodominant in Pfs230 and cleavage of this region may act as an immune evasion strategy of the parasite [6]. The proposed cleavage site in Pfs230 has been mapped (to between aa 477–487 and aa 523–555) using peptide specific antibodies but is not conserved among other species [6,7]. It is unknown if processing of Pfs230 is necessary and what role the prodomain plays for the protein function. While Pfs230 does not have a transmembrane domain, surface localization of Pfs230 on gametes is thought to be mediated in part as a complex with Pfs48/45, a glycosylphosphatidylinositol (GPI) anchored protein [5,9,10]. In *P. falciparum*, Pfs230-deficient males are unable to bind to red blood cells and establish exflagellation centers showing that Pfs230 is critical for fertilization and gamete fusion [11]. In *P. berghei*, Pbs230-deficient males fail to recognize female gametes [12].

Pfs230 is a member of the 6-cysteine (6-cys) protein family [13,14]. Thirteen other 6-cys proteins have been described in *P. falciparum*, namely Pfs230p (paralogue of Pfs230), Pf92, Pfs48/45, Pfs47, PfPSOP12, Pf52, Pf41, Pf38, Pf36, Pf12, Pf12p, PfB9, and PfLISP2, all of which are conserved across *Plasmodium* species and are differentially expressed throughout the life cycle [5,15]. This family of proteins contains between one to fourteen 6-cys domains which are often present as tandem pairs [13,14,16]. The 6-cys domain is a β-sandwich fold formed by two β-sheets of mixed parallel and antiparallel β-strands, with typically six disulfide-bonded cysteines that are positionally conserved [17]. The 6-cys domains with less than six cysteines are referred to as ‘degenerate’ 6-cys domains. Pfs230 is the largest 6-cys protein containing fourteen consecutive 6-cys domains, (D1 to D14, following the N-terminal prodomain. For Pfs230, the first 6-cys domain (D1) and part of the extended N-terminal sequence has been studied extensively as either a transmission blocking vaccine candidate or as a target of transmission blocking antibodies [18–23].

While clinical trials with Pfs230 D1 are ongoing (ClinicalTrials.gov Identifier: NCT02334462), there is not much known about the function of the other 6-cys domains within Pfs230. Epitope mapping of anti*-*Pfs230 antibodies suggests that only part of the prodomain and the D1 of Pfs230 can elicit transmission blocking antibodies [18,20,21]. To date, 20 inhibitory monoclonal antibodies (mAbs) specific for *P. falciparum* Pfs230 have been reported, which reduce the formation of oocysts in the mosquito midgut to varying degrees [15]. Of those, only crystal structures of the transmission blocking antibodies 4F12 and LMIV230-01 in complex with Pfs230 D1 and part of the prodomain have been determined [22,23].

We wanted to provide further structural insight into the interdomain structures between tandem Pfs230 domains and to determine if other domains of Pfs230 could elicit transmission blocking activity without the presence of the prodomain, which function remains unknown. Here we determined crystal structures of the first two 6-cys domains of Pfs230 in the presence and absence of a part of the prodomain. By screening a nanobody phage display library generated from an alpaca immunized with the first two domains of Pfs230, we identified 12 anti-Pfs230 nanobodies that bound at low nanomolar (nM) affinity and to two distinct epitope groups. Several anti-Pfs230 nanobodies inhibited exflagellation center formation and significantly reduced oocyst formation in the mosquito. Crystal structures of Pfs230 D1 in complex with inhibitory nanobodies provide structural insight into the mechanisms of inhibition.

## Results

### Crystal structures of the first two 6-cys domains of Pfs230

**Figure 1.**
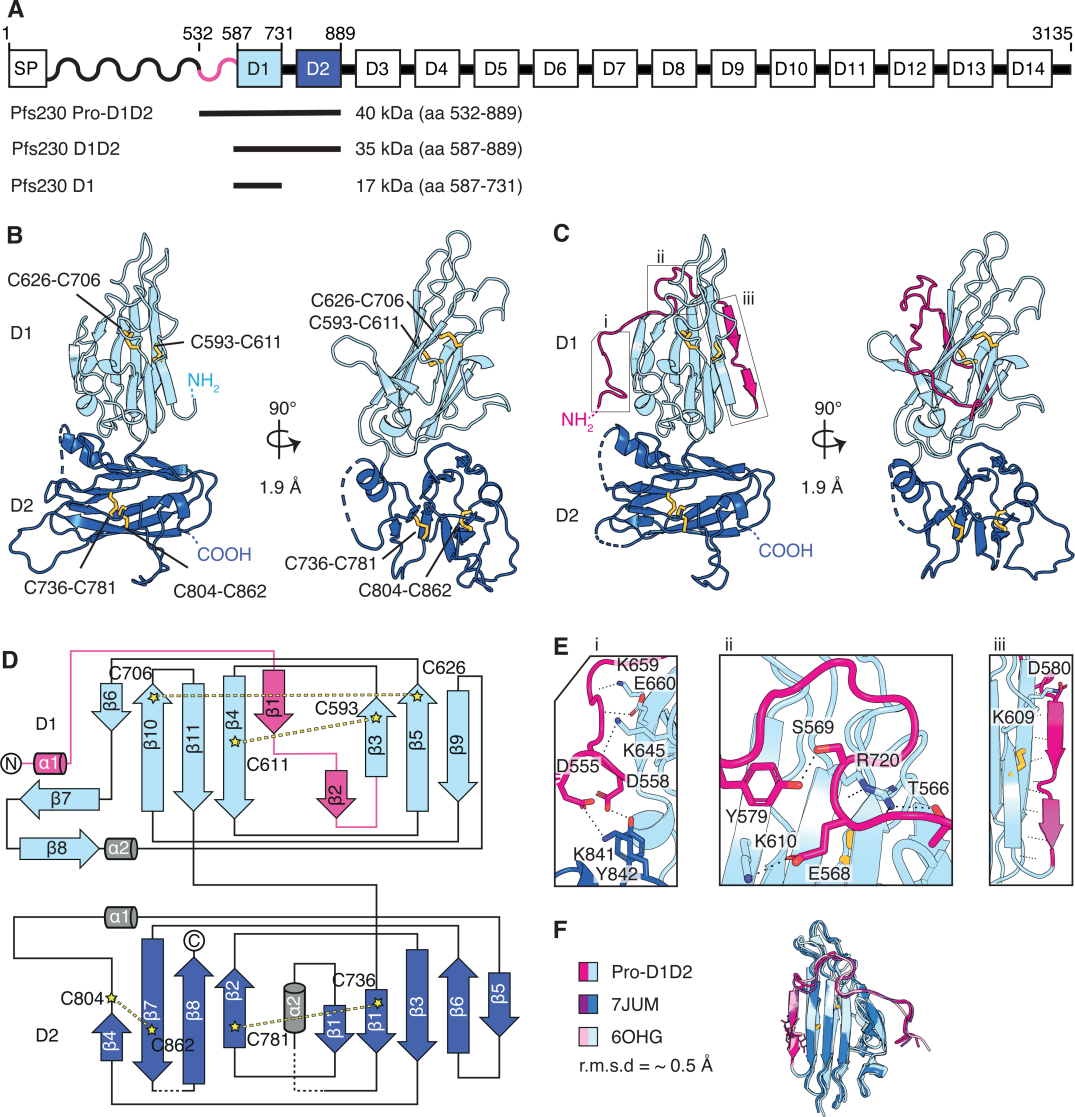
Crystal structures of Pfs230 D1D2 in the presence and absence of pro-domain residues. (**A**) Schematic diagram of Pfs230 and recombinant protein constructs. Signal peptide (SP), prodomain (wave) and 14 6-cys domains denoted from D1–D14 are indicated. (**B**) Crystal structure of Pfs230 D1D2 in two orthogonal views. N- and C-termini and disulfide bonds (yellow) are indicated. The D1 domain is shown in light blue and D2 domain in dark blue. Disulfide bonds are shown in ball and stick representation. (**C**) Crystal structure of Pfs230 Pro-D1D2 in two orthogonal views. Prodomain residues are shown in pink. Disulfide bonds (yellow) are shown in ball and stick representation. (**D**) Topology diagram of Pfs230 Pro-D1D2 is colored similarly to the schematic representation in Figure 1C. The disulfide bonds C593–C611 and C626–C706 are indicated in D1, and disulfide bonds C736–C781 and C804–C862 in D2. (**E**) Interactions between prodomain residues (pink) with residues of the 6-cys domains D1 and D2. Panels i to iii refer to the corresponding regions in Figure 1C. (**F**) Alignment of the D1 domain of Pfs230 Pro-D1D2 with Pfs230 of PDB ID 6OGH and 7JUM.

Full length Pfs230 is a 3135 amino acid (aa) protein that includes a signal peptide, a prodomain and fourteen 6-cys domains (labeled D1 to D14) (Figure 1A). To elucidate the structure of the first two 6-cys domains of Pfs230, we expressed Pfs230 D1D2 (aa 587–889) (Supplementary Fig. S1A), which contains the first two 6-cys domains and Pfs230 Pro-D1D2 (aa 532–889) (Supplementary Fig. S1A), which has a part of the prodomain and the first two 6-cys domains (Figure 1A). We determined the crystal structure of Pfs230 D1D2 by single isomorphous replacement with anomalous scattering (Figure 1B and Table 1) and used it to solve the phase problem of Pfs230 Pro-D1D2 by molecular replacement (Figure 1C and Table 1). Both crystal structures were determined at a resolution of 1.9 Å. The two proteins fold into two degenerate 6-cys domains, D1 and D2, each containing four cysteines.

**Table 1.** Data collection and refinement statistics for Pfs230 D1D2 and Pfs230 Pro-D1D2

	Pfs230 D1D2		Pfs230 Pro-D1D2
PDB ID		7USR	7USS
**Data collection statistics**	PIP-soaked	Native	
Wavelength (Å)	1.053401	0.968625	0.953732
Space group	P4_3_	P4_3_	P1
Cell axes (Å) (a, b, c)	65.65, 65.65, 107.98	65.79, 65.79, 108.04	35.25, 50.85, 108.97
Cell angles (°) (α, γ, β)	90, 90, 90	90, 90, 90	99.19, 98.86, 94.66
Resolution range (Å)	46.52–1.90 (1.95–1.90)	46.52–1.93 (2.04–1.93)	48.54–1.90 (2.01–1.90)
Completeness (%)	100.0 (100.0)	99.9 (99.5)	97.3 (94.9)
Total no. of reflections	500793 (71081)	492491 (79050)	205972 (31105)
Unique reflections	71088 (5262)	34715 (5565)	56540 (8901)
Redundancy	7.0 (6.8)	14.2 (14.2)	3.6 (3.5)
*R*_meas_ (%)	9.6 (109.2)	14.4 (149.4)	10.2 (87.2)
*CC*_1/2_ (%)	99.9 (75.3)	99.9 (73.2)	99.7 (68.5)
*I*/*σ*	13.59 (1.61)	16.64 (1.93)	9.44 (1.57)
Wilson B (Å^2^)	37.44	34.76	35.30
**Phasing**			
Resolution bin (Å)	46.52–3.50		
Sites per ASU	3		
*CC*_all_/*CC*_weak_ (%)	28.08/ 20.67		
Figure of merit	0.621		
**Refinement statistics**			
*R*_work_/*R*_free_ (%)		16.4/20.5	17.7/20.7
No. of atoms			
Protein		2320	4942
Water		298	377
Ions			2
B factors (Å^2^)			
Chain A		39.6	39.8
Chain B			42.3
Chain C			
Chain D			
Water		45.6	42.1
Ions			92.7
R.m.s. deviations			
Bond lengths (Å)		0.017	0.008
Bond angles (°)		1.300	0.892
Validation			
Ramachandran plot			
Outliers (%)		0.4	0.0
Favored (%)		97.9	97.9
Rotamer outliers (%)		0.8	0.2
C-beta outliers		0	0
MolProbity score		1.19	1.22

A single crystal was used for each data set.

PIP: Di-µ-iodobis(ethylenediamine)diplatinum(II)nitrate.

The values in parentheses represent the highest-resolution shell.

For Pfs230 D1D2 one molecule is present in the asymmetric unit with all residues defined except for 13 residues of a loop within the D2 domain and two residues at the C-terminus (Figure 1B). The D1 domain of Pfs230 D1D2 folds into a three-on-four β-sandwich and as typical for A-type 6-cys domains, the first two β-strands are pinned together by a disulfide bond between C593 and C611. A second disulfide bond is formed by C626 and C706 (Figure 1B,D). A short linker region connects the Pfs230 D1 domain with the D2 domain, which folds into a B-type 6-cys domain with disulfide bonds at C736–C781 and C804–C862 (Figure 1B,D). The orientation of the two domains towards each other is similar to tandem pairs of other 6-cys proteins such as Pf12, Pf41 and Pf12p, and is stabilized by several inter-domain contacts (Supplementary Fig. S2).

For Pfs230 Pro-D1D2, we observed two molecules in the asymmetric unit, which align well with each other and with Pfs230 D1D2 with root mean square deviation (r.m.s.d.) values between 0.234 and 0.390 Å (over 1876 and 1673 atoms, respectively). There are 15 residues of the N-terminus missing in the electron density of the Pfs230 Pro-D1D2 molecules. In contrast with Pfs230 D1D2, the D1 domain of Pfs230 Pro-D1D2 assembles into a four-on-five β-sandwich (Figure 1C,D) in which the prodomain residues wind around the core structure of the D1 domain and contribute one β-strand (labeled iii) to each leaf of the β-sandwich (Figure 1C–E). In comparison with previously published structures of Pfs230 D1 that contain an extended N-terminus (PDB ID 6OHG and 7JUM), the prodomain residues adopt a similar arrangement and form 15 hydrogen bonds and two salt bridges with residues of the D1 core structure (Figure 1E (i), (ii) and (iii), F). In our crystal structures, we also observed two prodomain residues that interact with the D2 domain; D555 forms a salt bridge with K841 and D558 interacts with Y842 (Figure 1E, panel i).

### High affinity nanobodies against Pfs230

**Figure 2.**
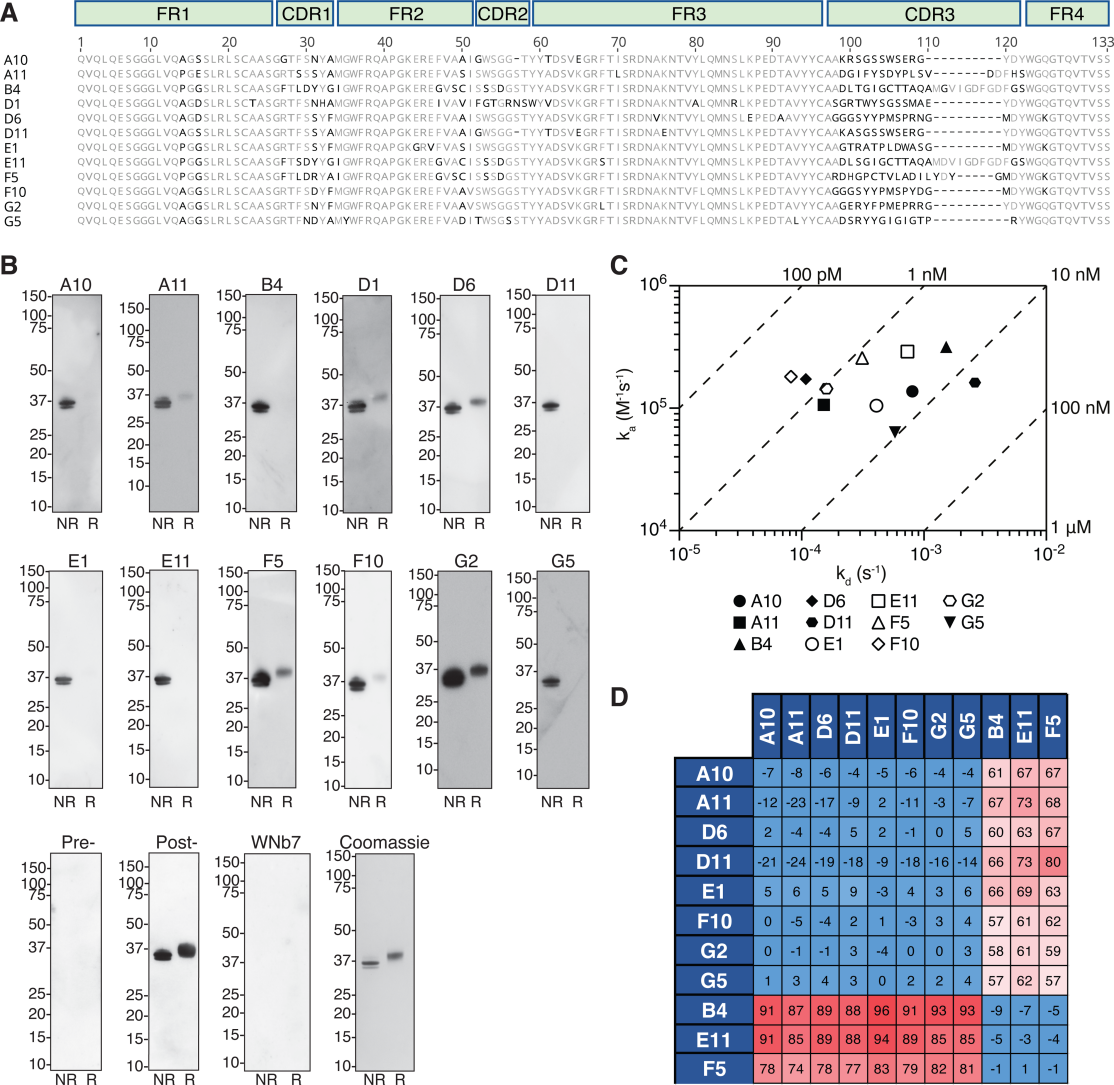
Pfs230 specific nanobodies. (**A**) Amino acid sequence alignment of 12 Pfs230 nanobodies with framework regions (FR) and complementary-determining regions (CDR) indicated. (**B**) Detection of recombinant Pfs230 D1D2 by nanobodies using Western blotting. Non-reduced (NR) and reduced (R) Pfs230 D1D2 protein was separated by SDS–PAGE and probed with the respective nanobodies (top and middle row), pre- and post-immunization alpaca sera, or a SARS-CoV-2 nanobody WNb7 (bottom row). An HRP-conjugated goat anti-llama IgG was used for detection. Molecular mass marker (M) in kDa is shown on the left-hand side. A Coomassie-stained SDS–PAGE gel is used as a loading control (bottom row, right hand-side). (**C**) Iso-affinity plot showing the dissociation rate constants (*k*d) and association rate constants (*k*a) of nanobody binding to Pfs230 D1D2 as measured by BLI. Data points are the average of two independent experiments. Diagonal lines indicate equilibrium dissociation rate constants (*K*_D_). (**D**) Epitope competition experiments with immobilized nanobodies indicated on the left column incubated with nanobodies indicated on the top row pre-incubated with Pfs230 D1D2 using a 10 : 1 molar ratio. Percentage binding of Pfs230 D1D2 pre-incubated with nanobody was calculated relative to Pfs230 D1D2 binding alone, which was assigned to 100%. The red and blue boxes represent non-competing and competing nanobodies, respectively.

To identify nanobodies against Pfs230, we immunized an alpaca with recombinant Pfs230 D1D2 and generated a 10^6^ nanobody phage display library. After two rounds of phage display, we identified 12 distinct nanobody clonal groups based on differences in the amino acid sequence of the complementary determining region 3 (CDR3) that vary in at least one amino acid (Figure 2A and Supplementary Table S1). The CDR3 regions of the nanobodies vary in length between 14–24 residues (Figure 2A). One member of each clonal group was purified and referred to as A10, A11, B4, D1, D6, D11, E1, E11, F5, F10, G2, and G5, and nanobodies migrated between 14 and 16 kDa under reducing conditions (Figure 2A and Supplementary Fig. S1B).

All 12 nanobodies detected recombinant Pfs230 D1D2 under non-reducing conditions, with F5 and G2 showing the strongest signal (Figure 2B). Nanobodies A10, B4, D11, E1, E11 and G5 showed no reactivity for reduced Pfs230 D1D2, while A11, D1, D6, F5, F10, and G2 recognized Pfs230 D1D2 under reducing conditions but to a lesser extent compared with non-reducing conditions (Figure 2B). As a negative control, a SARS-CoV-2 spike nanobody, WNb7, showed no reactivity for Pfs230 D1D2 under both conditions [24]. Pre-immunization polyclonal alpaca serum showed no reactivity to Pfs230 D1D2, whereas as expected, post-immunization serum showed strong detection of Pfs230 D1D2 (Figure 2B).

Using bio-layer interferometry (BLI), we determined the binding affinities of the nanobodies against Pfs230 D1D2 (Figure 2C and Supplementary Fig. S3). Binding affinities to Pfs230 D1D2 in the nanomolar range were observed for 11 of the 12 nanobodies, with equilibrium dissociation constants (*K*_D_) between 0.45 nM and 16.15 nM (Figure 2C and Supplementary Fig. S3A,C). No binding was detected between nanobody D1 and Pfs230 D1D2. However, we observed that all 12 nanobodies bound to Pfs230 D1 (aa 587–731) with binding affinities comparable to Pfs230 D1D2 in the nanomolar range with equilibrium dissociation constants (*K*_D_) between 0.14 nM and 8.31 nM (Supplementary Fig. S3B,C). The ability of nanobody D1 to bind to Pfs230 D1 but not to Pfs230 D1D2 indicates that its binding epitope is not accessible in the folded form of Pfs230 D1D2.

We performed a nanobody competition experiment to determine if the eleven Pfs230 D1D2-specific nanobodies bound to similar epitopes. As expected, all nanobodies were able to compete with themselves (Figure 2D). We observed that B4, E11 and F5 compete with each other, but not with A10, A11, D6, D11, E1, F10, G2, and G5. Collectively, we show that these Pfs230 nanobodies bind in two different epitope bins within the Pfs230 D1 domain. We focused our subsequent characterization on nanobodies F5 and F10, as they were the highest affinity binders from each of the epitope bins.

### Anti-Pfs230 nanobodies bind to gametocytes and block transmission

**Figure 3.**
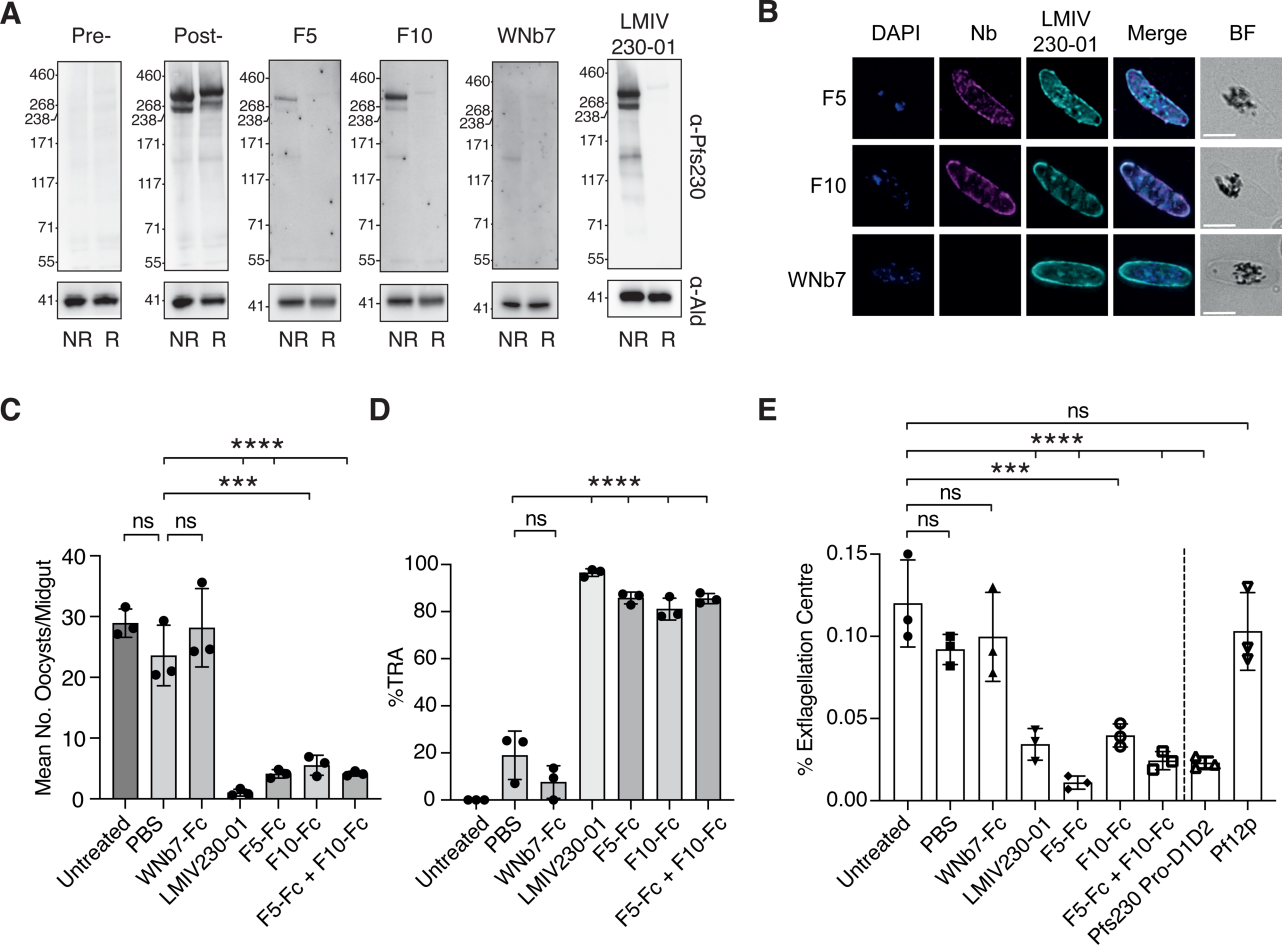
Anti-Pfs230 nanobodies recognize gametocytes and block transmission. (**A**) Detection of Pfs230 of stage V gametocytes by nanobodies using western blotting. For detection, 0.5 µg/ml nanobodies, 0.1 µg/ml LMIV230-01, and 1/500 alpaca serum pre- and post-immunization were used. (**B**) Immunofluorescence assay of stage V gametocytes stained with either the F5 or F10 nanobody (magenta) counterstained with the human antibody LMIV230-01 (cyan). DAPI stains the nucleus (blue). BF: bright field. Merge images are shown. Scale bar: 5 µm. (**C**) Standard membrane feeding assays with mean oocysts /dissected midgut of *A. stephensi* mosquitos seven days post feeding with 0.5% Stage V NF54 gametocytes. Untreated parasites were compared with treatment with either PBS control, nanobody-Fcs or LMIV230-01. For each experimental group, *n* = 20 mosquitos were dissected. (**D**) Calculated % Transmission Reduction Activity (% TRA) relative to the untreated control was obtained from results shown in Figure 3C. (**E**) Mean number of exflagellation centers relative to total number of red blood cells in untreated, PBS control, nanobody-Fcs, LMIV230-1, Pfs230 Pro-D1D2 and Pf12p -treated groups. Three independent experiments were performed for standard membrane feeding and exflagellation assays. Statistical significances were determined using one-way ANOVA and Tukey's multiple comparison test. **** *P* ≤ 0.0001. *** *P* ≤ 0.001. ns *P* > 0.05.

The F5 and F10 anti-Pfs230 nanobodies detected 350 kDa and 250 kDa bands in stage V gametocytes under non-reducing conditions (Figure 3A). The 350 kDa band corresponds to full-length Pfs230 protein and the 250 kDa band likely represents the proteolytically cleaved Pfs230. As positive controls, anti-Pfs230 mAb LMIV230-01 and post-immunization alpaca sera showed similar detection of the two bands [22]. As expected, pre-immunization alpaca sera and the WNb7 negative control did not detect Pfs230 in gametocytes. In addition, we observed that the binding of Pfs230 nanobodies F5 and F10 co-localizes with anti-Pfs230 mAb LMIV230-01 staining on the surface of stage V gametocytes (Figure 3B). These results suggest that nanobodies F5 and F10 recognize Pfs230 in the sexual stages of *P. falciparum*.

To determine if our Pfs230 nanobodies inhibit parasite transmission, we first expressed F5 and F10 as Fc-fusion proteins (F5-Fc and F10-Fc) to allow comparison to the transmission blocking mAb LMIV230-01 (Supplementary Fig. S1C) [22]. F5-Fc and F10-Fc bound to Pfs230 D1D2 with binding affinities equivalent to monomeric nanobodies and *K*_D_ values of 0.57 nM and 0.13 nM, respectively (Supplementary Fig. S4). When *P. falciparum* gametocytes (strain NF54) were fed to *Anopheles stephensi* mosquitos in a standard membrane feeding assay, we observed an average of 28.92 ± 2.34 oocysts per midgut. The addition of PBS as a buffer control or 100 µg/ml WNb7-Fc did not significantly reduce the average oocysts number (23.59 ± 4.98 and 28.16 ± 6.45, respectively) (Figure 3C and Supplementary Table S2). In contrast, addition of 100 µg/ml F5-Fc or F10-Fc significantly reduced *P. falciparum* oocyst numbers to 4.11 ± 0.70 and 5.52 ± 1.66, respectively (Figure 3C). A cocktail of F5-Fc and F10-Fc did not increase their overall potency compared with the single entities with reduced oocyst numbers at 4.14 ± 0.39. Transmission blocking mAb LMIV230-01 at 200 µg/ml (equivalent molar concentration to the nanobody-Fc) significantly reduced mean oocyst numbers to 1.02 ± 0.57, as previously reported [22]. We showed that F5-Fc, F10-Fc and LMIV230-01 significantly reduced oocyst numbers by 83, 79, and 97%, respectively compared with the untreated control (Figure 3D).

Using an exflagellation center formation assay, we observed that the addition of F5-Fc, F10-Fc and LMIV230-01 reduced the numbers of exflagellation centers compared with our untreated, PBS or WNb7-Fc controls (Figure 3E). Untreated parasites have an average of 0.09–0.12% of exflagellation centers, whereas the addition of F5-Fc and F10-Fc reduced exflagellation centers to 0.01 ± 0.004% and 0.04 ± 0.007%, respectively (Figure 3E). LMIV230-01 reduced the number of exflagellation centers to 0.03 ± 0.01%, while WNb-7-Fc showed no effect (Figure 3E). Furthermore, the addition of recombinant Pfs230 Pro-D1D2 protein at a concentration of 100 µg/ml reduced the number of exflagellation centers to 0.02 ± 0.004% while recombinant Pf12p, another 6-cys protein, showed no reduction at the same concentration (Figure 3E). These results show that anti-Pfs230 nanobodies can block *P. falciparum* parasite transmission.

### Crystal structures of transmission blocking nanobodies F5 and F10 in complex with Pfs230

**Figure 4.**
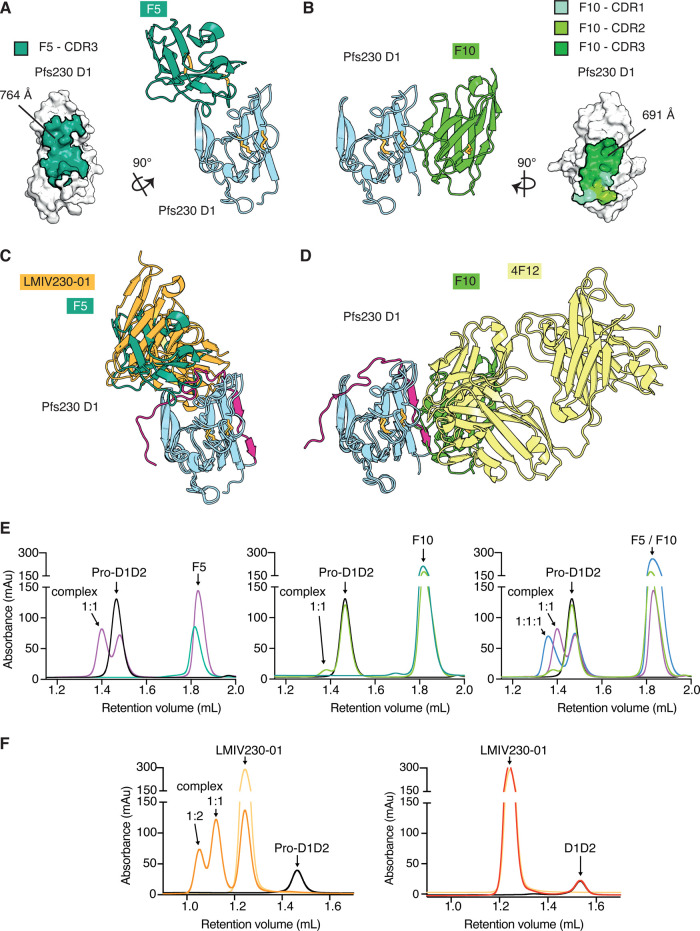
Crystal structures of Pfs230 D1 in complex with F5 and F10 nanobodies. (**A**) Crystal structure of Pfs230 D1 bound to nanobody F5. Left, surface representation of Pfs230 D1 with the epitope of F5 colored in green. Only CDR3 forms contacts with Pfs230 D1. Right, ribbon representation showing Pfs230 D1 in blue and F5 in green. (**B**) Left, crystal structure of Pfs230 D1 (blue) bound to nanobody F10 (light green). Right, surface representation of Pfs230 D1 with the epitope of F10 colored in light (CDR1), medium (CDR2) and dark green (CDR3). (**C**) Alignment of the complex between Pfs230 D1 (blue) and F5 (green) with PDB ID 7JUM. Pfs230 is shown in blue except for residues of the pro-domain region which are shown in pink. The single-chain fragment variable of transmission blocking mAb LMIV230-01 is shown in orange. (**D**) Alignment of the Pfs230 D1 (blue) — F10 (light green) complex with PDB ID 6OHG. The Fab fragment of transmission blocking mouse mAb 4F12 is shown in yellow. (**E**) Size exclusion chromatography (SEC) of Pfs230 Pro-D1D2 with nanobodies. SEC of individual proteins are shown in addition, with Pfs230 Pro-D1D2 in black and nanobodies in dark green. Pfs230 Pro-D1D2 with F5 (left) in a 1 : 2 molar ratio (purple). Pfs230 Pro-D1D2 with F10 (middle) in a 1 : 2 molar ratio (light green). Pfs230 Pro-D1D2 with F5 and F10 (right) in a 1 : 2 : 2 molar ratio (blue line). For comparison, SEC of Pfs230 Pro-D1D2 with F5 or F10 alone in a 1 : 2 molar ratio as indicated. (**F**) SEC of recombinant Pfs230 with LMIV230-01 as follows: (left) LMIV230-01 alone (light orange), Pfs230 Pro-D1D2 (black), and a 2 : 1 molar mix of LMIV230-01 with Pfs230 Pro-D1D2 (dark orange). (Right) Pfs230 D1D2 alone (black), LMIV230-01 alone (light orange), and a 2 : 1 molar mix of LMIV230-01 with Pfs230 D1D2 (red).

We determined crystal structures of anti-Pfs230 F5 and F10 nanobodies in complex with Pfs230 D1 to 1.7 and 2.1 Å resolution, respectively (Figure 4A,B, and Table 2). As expected, the crystal structures show that F5 and F10 nanobodies bind to different sites on Pfs230 D1. The F5 nanobody faces one β-sheet of the Pfs230 D1 β-sandwich. The CDR3 of F5, which is stabilized by an internal disulfide bond, forms most interactions with Pfs230 D1 with an extensive buried surface area of 764 Å^2^ (Figure 4A and Supplementary Table S3). For F5, it appears that CDR1 is not involved in contacting Pfs230 and several residues of CDR2 are not resolved in the structure. On the other hand, F10 binds to one side of the Pfs230 D1 β-sandwich and forms contacts with residues of the first two β-strands and β-strand connecting loops (Figure 4B). Here, all three CDR loops in F10 are involved in interactions with Pfs230 D1, forming an extensive buried surface area of the epitope with 691 Å^2^ (Figure 4B and Supplementary Table S4).

**Table 2.** Data collection and refinement statistics for Pfs230 D1 — nanobody complexes

	Pfs230 D1 — F5	Pfs230 D1 — F10
PDB ID	7UST	7USV
**Data collection statistics**		
Wavelength (Å)	0.953732	0.953732
Space group	C222_1_	P2_1_2_1_2_1_
Cell axes (Å) (a, b, c)	42.30, 84.18, 184.88	48.08, 101.22, 148.18
Cell angles (°) (α, γ, β)	90, 90, 90	90, 90, 90
Resolution range (Å)	42.09–1.70 (5.07–1.70)^1^	47.89–2.10 (2.22–2.10)^1^
Completeness (%)	99.9 (99.2)	99.5 (96.9)
Total no. of reflections	445981 (72424)	422617 (65033)
Unique reflections	36891 (5877)	43435 (6897)
Redundancy	12.1 (12.4)	9.7 (9.4)
*R*_meas_ (%)	5.0 (104.7)	24.2 (143.4)
*CC*_1/2_ (%)	100.0 (82.9)	98.0 (68.3)
*I*/*σ*	24.85 (2.17)	11.83 (1.76)
Wilson B (Å^2^)	39.34	40.78
**Refinement statistics**		
*R*_work_/*R*_free_ (%)	18.9/22.0	19.8/23.5
No. of atoms		
Protein	2105	5221
Water	118	349
Ions	15	40
B factors (Å^2^)		
Chain A	42.5	47.8
Chain B	60.2	50.8
Chain C		34.2
Chain D		36.5
Water	50.4	47.5
Ions	83.3	77.7
R.m.s. deviations		
Bond lengths (Å)	0.014	0.003
Bond angles (°)	1.261	0.585
Validation		
Ramachandran plot		
Outliers (%)	0.0	0.0
Favored (%)	98.1	98.7
Rotamer outliers (%)	0.0	0.0
C-beta outliers	0	0
MolProbity score	1.21	1.06

1 The values in parentheses represent the highest-resolution shell.

An overlay of the structural coordinates of our Pfs230 D1-nanobody structures, which do not contain prodomain residues, with previously published Pfs230 structures PDB ID 6OHG and 7JUM, shows that both F5 and F10 nanobodies bind to Pfs230 regions that usually contact residues of the prodomain (pink, Figure 4C,D). This finding suggests that F5 and F10 nanobodies will most likely compete with the prodomain for binding to Pfs230. In contrast, known transmission blocking LMIV230-01 and 4F12 mAbs bind to Pfs230 with some interactions between prodomain and mAb residues [22,23,25]. The F5 nanobody binds to a similar region on Pfs230 as LMIV230-01 [22], whereas F10 nanobody engages a similar region on Pfs230 to 4F12 (Figure 4C,D) [23,25].

To determine if the F5 and F10 nanobodies compete for Pfs230 binding with the prodomain, we performed a size shift assay using size exclusion chromatography (Figure 4E,F and Supplementary Fig. S5). Both nanobodies were able to complex some Pfs230 Pro-D1D2, as shown by a partial shift of the recombinant proteins to a higher molecular weight complex, which corresponds to a 1 : 1 complex of nanobody and Pfs230 Pro-D1D2 (Figure 4E, left and middle panel, and Supplementary Fig. S5). At the same molar excess, nanobody F5 appeared to complex more readily to Pfs230 Pro-D1D2 than nanobody F10. With an increasing molar excess of nanobody F5, more complex was formed but this trend did not further increase at a molar ratio of 4 : 1 (Supplementary Fig. S5A). The binding of F5 and F10 simultaneously to Pfs230 Pro-D1D2 resulted in a molecular weight shift that corresponds to a 1 : 1 : 1 complex. It appears that the combination of F5 and F10, permits F10 to bind more efficiently to Pfs230 Pro-D1D2 compared with the binding of the single entity (Figure 4E, right panel). We tested LMIV230-01 for binding to Pfs230 Pro-D1D2 and Pfs230 D1D2 (Figure 4F). As expected, LMIV230-01 formed a higher molecular weight complex with Pfs230 Pro-D1D2 with all of Pfs230 Pro-D1D2 shifting to higher molecular weights indicating complete complex formation with LMIV230-01 (Figure 4F, left panel). In contrast, LMIV230-01 did not form a complex with Pfs230 D1D2 showing that its extensive interactions with prodomain residues are critical for its binding to Pfs230 (Figure 4F, right panel).

Furthermore, we tested the binding of Pfs230 D1D2 and Pfs230 Pro-D1D2 to F5, F10 and LMIV230-01 using BLI. Consistent with our size shift assay results, we detected binding between immobilized F5-Fc and F10-Fc with Pfs230 Pro-D1D2. However, the binding of both nanobody-Fcs to Pfs230 Pro-D1D2 was significantly weaker than their binding to Pfs230 D1D2 (Supplementary Fig. S4). The affinity of F5-Fc to Pfs230 Pro-D1D2 (117.50 nM) was 200-fold weaker than its affinity to Pfs230 D1D2 (0.57 nM), which was mostly caused by a slower on-rate (Supplementary Fig. S4). The affinity of F10-Fc to Pfs230 Pro-D1D2 (632.50 nM) was also weaker than its affinity to Pfs230 D1D2 (0.13 nM), and a result of a slower on-rate (Supplementary Fig. S4). Similar to the size shift assay results, F10-Fc shows weaker binding to Pfs230 Pro-D1D2 compared with F5-Fc and has an even slower on-rate (Supplementary Fig. S4). The binding affinity of LMIV230-01 with Pfs230 Pro-D1D2 is 3.98 nM and we did not observe any binding to Pfs230 D1D2, even at a high Pfs230 D1D2 concentration of 3000 nM. These results suggest that F5 and F10 compete with the prodomain and dislodge it before binding to Pfs230 Pro-D1D2.

## Discussion

Here, we present the first crystal structures of two domains of Pfs230 in the presence and absence of a part of the prodomain. Immunization of an alpaca with the first two 6-cys domains of Pfs230 enabled the identification of high affinity nanobodies against Pfs230 (*K*_D_ ranging from 0.14 nM and 14.05 nM). Using X-ray crystallography and BLI, we show that nanobodies F5 and F10 bind to distinct epitopes on Pfs230 D1. These nanobodies fused to an Fc domain showing significant reduction in *P. falciparum* transmission and exflagellation center formation. To the best of our knowledge, these are the first nanobody-Fc fusions that bind Pfs230 D1 in the absence of the prodomain that block malaria parasite transmission.

Pfs230 is composed of fourteen 6-cys domains. To date, the crystal structures of Pfs230 predominantly feature D1 and part of the prodomain. We present the first crystal structures describing the Pfs230 D1D2 domains and show that the tandem pair of degenerate 6-cys domains adopt a similar organization to typical 6-cys domain pairs such as in Pf12, Pf41 and Pf12p [16,26,27] (Figure1 and Supplementary Fig. S2). In our Pfs230 Pro-D1D2 structure, we observed that prodomain residues add additional β-strands to the D1 core structure and form contacts with the D1 domain as previously described [22,23]. We identified additional contacts between the prodomain and the Pfs230 D2 domain (Figure 1C,E, panel i), which were not observed in either previous crystal structures or an AlphaFold prediction of full-length Pfs230 [15]. In addition, our Pfs230 D1D2 structure highlights that the Pfs230 D1 domain is stable and well-folded without the prodomain.

Our transmission blocking nanobodies F5 and F10 bind to Pfs230 in the absence of the prodomain. An overlay of the nanobody structures with crystal structure of Pfs230 D1 containing prodomain residues suggests that the nanobodies would compete with the prodomain for binding to Pfs230 (Figure 4C,D). This is a different mechanism of binding of other known transmission blocking antibodies such as LMIV230-01 and 4F12, which bind Pfs230 in the presence of the prodomain with several antibody-prodomain interactions observed in the crystal structures [23]. From our transmission blocking experiments, LMIV230-01 is more potent than either of our nanobody-Fc fusions (Figure 3C). One potential explanation for the reduction in transmission blocking potency is that F5 and F10 have to compete with the prodomain for binding efficiently to Pfs230 D1 whereas LMIV230-01 only binds Pfs230 in the presence of the prodomain. Pfs230 and nanobody complex formation assays show that nanobody F5 is more efficient in dislodging the prodomain for binding to Pfs230 Pro-D1D2 than nanobody F10. In presence of F5, F10 binding to Pfs230 Pro-D1D2 seemed increased compared with the single nanobody binding event (Figure 4E, right panel). It would be interesting to observe if an F5 and F10 biparatopic-Fc fusion, which may allow simultaneous binding, would be more effective at blocking parasite transmission compared with LMIV230-01 and 4F12. On the other hand, future transmission blocking experiments that combine antibodies/nanobodies that bind in the presence and absence of the prodomain would shed light on whether these combinations of antibodies have increased potency in transmission blocking.

Transmission blocking nanobody F5 binds to a similar face on Pfs230 as LMIV230-01 whereas nanobody F10 binds to a similar face to 4F12 (Figure 4C,D). LMIV230-01 is a human monoclonal antibody isolated from single memory B cells from Malian adults immunized with four doses of Pfs230D1-EPA/Alhydrogel® (Clinicaltrials.gov NCT02334462). 4F12 is a mouse monoclonal antibody obtained from hybridomas by immunizing mice with enriched preparations of *P. falciparum* female gametocytes and was subsequently observed to recognize recombinant Pfs230 protein [25]. The 12 nanobodies which were isolated using phage display methods, bound to only two major epitope sites. It may be that these two sites, which are also recognized by human and mouse antibodies and nanobodies are more immunogenic compared with other sites within Pfs230 D1. Previously, immunization of alpacas with Pf12p D1D2 allowed the identification of nanobodies that bound to the interdomain region between D1 and D2 [26]. Furthermore, immunization with Pf12 D1D2 did identify nanobodies that bound only to Pf12D2 that blocked complex formation with its binding partner Pf41 [27]. Further work to identify different classes of Pfs230 nanobodies may provide alternative mechanisms to block *P. falciparum* transmission.

Pfs230 is known to be present on the surface of male and female gametocytes. While it does not have a transmembrane domain, it binds to Pfs48/45 which has a GPI anchor, another 6-cys protein and a critical protein during malaria parasite sexual stages [9,10]. Pfs48/45 is part of several multi-antigen transmission blocking vaccine candidates and in particular, the Pfs230–Pfs48/45 chimera elicited transmission blocking antibody responses three-fold higher than the single antigens alone [23]. More recently, administration of the humanized monoclonal antibody against Pfs48/45, TB31F at 10 mg/kg, was found to be well-tolerated in a Phase 1 clinical trial and participant sera showed >80% transmission reducing activity (TRA) for more than 84 days [28]. Future development of transmission blocking monoclonal antibody interventions may consider the design of bispecific antibodies to multiple transmission blocking antigens or antibody cocktail combinations that may result in reduced dosages and/or prevent the emergence of resistance mutants.

While crystal structures for the first two domains of Pfs230 are now available, there is still no experimental structural insight into full-length Pfs230 which is mostly composed of fourteen 6-cys domains. Previous studies of multiple Pfs230 6-cys domains have shown that only Pfs230 D1 elicits transmission blocking antibodies [20]. Unfortunately, our attempts to obtain nanobodies to Pfs230 D2 were also not successful. Since our phage display approach immobilizes the antigen onto a charged plate surface, this may have resulted in a misfolding of D2. In the future, we may consider using site-specific biotinlyation to allow presentation of the antigen on the surface of streptavidin beads which may reduce misfolding of Pfs230 D2.

However, until Pfs230 is expressed in its entirety, it would be difficult to rule out that the other thirteen 6-cys domains are not important in the critical functions of Pfs230 during the malaria parasite sexual stages and successful fertilization. Similarly, the C-terminal domain of Pfs48/45 was thought to be the major contributor to transmission blocking antibodies. However, the recent successful expression of the full Pfs48/45 ectodomains show that the N-terminal and central domains also contribute substantially to the transmission blocking activity in immunized animals [29].

Our structural characterization of Pfs230 D1D2 and the first collection of nanobodies against Pfs230 provide us with an improved understanding of one of the leading transmission blocking vaccine candidates. Furthermore, transmission blocking nanobodies show a different mode of binding compared with known transmission blocking antibodies of Pfs230. Future studies exploring combinations of nanobodies against multiple antigens involved in *P. falciparum* sexual stage development and fertilization may assist in delivering a more potent antibody intervention to block malaria transmission.

## Methods

### Pfs230 recombinant protein expression and purification

We expressed and purified fragments of Pfs230 (PF3D7_0209000) that correspond to amino acids 587-731 (Pfs230 D1), 587-889 (Pfs230 D1D2), and 532-889 (Pfs230 Pro-D1D2). Codon optimized (*Spodoptera frugiperda, Sf*) DNA was cloned into a modified form of baculovirus transfer vector pAcGP67-A. The Pfs230 sequence is in frame with the GP67-signal sequence, an octa-histidine tag, and a Tobacco Etch Virus (TEV) protease cleavage site. Pfs230 proteins were produced using Sf21 cells (Life Technologies) and Insect-XPRESS Protein-free Insect Cell Medium supplemented with l-glutamine (Lonza). A cell culture of ∼1.8 × 10^6^ cells/ml was inoculated with the third passage stock of virus and incubated for three days at 28°C. Cells were separated from the supernatant by centrifugation at 7000×***g*** for 20 min. The supernatant was sterile filtered with 0.45 µm filters and concentrated via tangential flow filtration using a 10 kDa molecular weight cut-off cassette (Millipore). The concentrated supernatant was sterile filtered 0.45 µm filter and dialyzed into 30 mM Tris pH 7.5, 300 mM NaCl (buffer A). The dialyzed sample was incubated with Ni-NTA resin (Qiagen) for 1 h, at 4°C on a roller shaker. A gravity flow chromatography column was washed with 10–20 column volumes of buffer A, as well as with stepwise increased imidazole concentrations in buffer A. The different Pfs230 protein fragments eluted at an imidazole concentration of ∼70 mM. TEV protease was added to the pooled fractions containing Pfs230, while dialyzing into buffer A, to remove the N-terminal tag. The solution was incubated with Ni-NTA resin (Qiagen) for 1 h, 4°C on a roller shaker before untagged Pfs230 was separated from His-tagged TEV protease and un-cleaved protein via Ni-IMAC purification. The flow-through was concentrated and applied onto a SD200 increase 10/300 size exclusion chromatography column (Cytiva) pre-equilibrated with 20 mM HEPES pH 7.5, 150 mM NaCl.

### Nanobody and nanobody-Fc fusions expression and purification

Nanobodies were expressed in *Escherichia coli* WK6 cells. Bacteria were grown in Terrific Broth supplemented with 0.1% (w/v) glucose and 100 µg/ml carbenicillin at 37°C to an OD_600_ of 0.7, induced with 1 mM IPTG and grown overnight at 28°C. Cell pellets were harvested and resuspended in 20% (w/v) sucrose, 20 mM imidazole pH 7.5, 150 mM NaCl DPBS and incubated for 15 min on ice. An amount of 5 mM EDTA pH 8.0 was added and incubated on ice for 20 min. After this incubation, 10 mM MgCl_2_ was added, and periplasmic extracts were harvested by centrifugation and the supernatant was loaded onto a 1 ml HisTrap FF column (GE Healthcare). The nanobodies were eluted with 400 mM imidazole pH 7.5, 100 mM NaCl, PBS, subsequently concentrated and buffer exchanged into 20 mM HEPES pH 7.5, 150 mM NaCl.

Nanobody sequences of F5 and F10 were subcloned into a derivative of pHLSec containing the hinge and Fc region of human IgG1 to produce nanobody-Fc tagged fusion proteins in HEK293 cells via transient transfection. The supernatant was harvested six days after transfection and applied onto HiTrap PrismA affinity columns (Cytiva). Fusion proteins were eluted in 100 mM citric acid pH 3.0 and neutralized by the addition of 1 M Tris–HCl pH 9.0. Size exclusion chromatography in HT-PBS was used as last purification step.

### Expression and purification of LMIV230-01

The variable regions of the heavy and light chain of anti-Pfs230 mAb LMIV230-01 [22] were cloned into AbVec-hIgG1 (using AgeI and SalI restriction enzyme sites) and AbVec-IgKappa vectors (Addgene) (using AgeI and BsiWI restriction enzyme sites), respectively. LMIV230-01 was produced in HEK293 cells via transient transfection using a 1 : 1 ratio of light and heavy chain plasmids and purified following the same protocol that was used for nanobody-Fc fusion proteins as described above.

### Crystallization and structure determination

For crystallization of Pfs230 D1D2 and Pfs230 ProD1D2, the Collaborative Crystallization Center (CSIRO, C3, Parkville) was used. Pfs230 D1D2 crystals grew with 6.8 and 2.8 mg/ml protein at 8°C within a few hours. In one sparse matrix crystallization screen, crystallization hits were obtained in 65 different conditions. Crystals grown in 20% (w/v) PEG 3350, 0.2 M sodium formate were harvested with 30% glycerol in mother liquor. For experimental phasing one crystal grown in 16% (w/v) PEG 3350, 0.2 M sodium formate at 8°C via the hanging drop vapor diffusion method was soaked in mother liquor containing 3 mM di-µ-iodobis(ethylenediamine) diplatinum (II) nitrate (PIP) and 30% glycerol before flash freezing in liquid nitrogen.

An initial crystallization screen of Pfs230 Pro-D1D2 was set up and incubated with 10 mg/ml at 8°C. After three months the plate was transferred to 4°C and a crystal grew within one week in 30% (w/v) PEG MME 2000, 0.1 M potassium thiocyanate, and was harvested with 25% glycerol in mother liquor. Crystallization screens of Pfs230 D1 in complex with nanobody F5 and F10, respectively, were set up at the Monash Macromolecular Crystallization Facility (MMCF, Clayton, VIC, Australia) with 3–10 mg/ml at 4°C. Crystallization conditions were optimized in-house via the hanging drop vapor diffusion method. Pfs230 D1- F5 crystals grew in 2.0 M ammonium Sulfate, 0.1 sodium citrate pH 5.5 and Pfs230 D1- F10 crystals appeared in 0.2 M ammonium Sulfate, 0.1 M sodium acetate pH 4.6, and 25% (w/v) PEG 4000.

X-ray diffraction data was collected at the beamlines MXI and MXII of the Australian Synchrotron. The XDS package (Kabsch, 2010) was used for data processing. The phase problem of Pfs230 D1D2 was solved with hkl2map and shelxCDE [30,31] using SIRAS phasing. Anomalous data was included to a resolution of 3.5 Å and native data to 1.93 Å. Molecular replacement was used to solve the phase problem of Pfs230 Pro-D1D2, Pfs230 D1- F5 and Pfs230 D1- F10 using structural coordinates of Pfs230 D1D2 and nanobody D9 of PBD ID 7KJI. Iterative cycles of structure building and refinement was carried out using *Coot* [32] and Phenix [33,34]. Figures of the structure were prepared with PyMOL [35]. The atomic coordinates and structure factor files have been deposited in the Protein Data Bank under PDB ID 7USR (Pfs230 D1D2), 7USS (Pfs230 Pro-D1D2), 7UST (Pfs230 D1- F5) and 7USV (Pfs230 D1- F10).

### Isolation of nanobodies against Pfs230

One alpaca was immunized six times with ∼200 µg of recombinant Pfs230 D1D2 and GERBU FAMA as adjuvant. Immunization and handling of the alpaca for scientific purposes was approved by Agriculture Victoria, Wildlife & Small Institutions Animal Ethics Committee, project approval No. 26-17. Blood was collected three days after the last immunization for the preparation of lymphocytes. Nanobody library construction was carried out according to established methods [36]. Briefly, alpaca lymphocyte mRNA was extracted and amplified by RT-PCR with specific primers to generate a cDNA library size of 10^6^ nanobodies with 80% correctly sized nanobody insert. The library was cloned into a pMES4 phagemid vector amplified in *E. coli* TG1 strain and subsequently infected with M13K07 helper phage for recombinant phage expression. Phage display to identify anti-Pfs230 nanobodies was performed as previously described with the following modifications [36]. Phages displaying Pfs230-specific nanobodies were enriched after two rounds of biopanning on 1 µg of immobilized Pfs230 D1D2. After the second round of panning, 95 individual clones were selected for further analyses by ELISA to identify positive nanobody clones that were specific against Pfs230 D1D2. Positive clones were sequenced and annotated using the International ImMunoGeneTics database (IMGT) and aligned in Geneious Prime [37].

### Nanobody affinities and epitope binning using BLI

Affinity determination measurements were performed on the Octet RED96e (FortéBio). Assays using monomeric nanobodies were performed using Ni-NTA capture sensor tips (Octet®NTA), while for assays using nanobody-Fc or LMIV230-01 anti-hIgG Fc capture sensor tips (Octet®AHC) were used. All measurements were performed in kinetics buffer (PBS pH 7.4 supplemented with 0.1% (w/v) BSA and 0.05% (v/v) TWEEN-20) at 25°C. After a 60 s baseline step, nanobodies, nanobody-Fcs or LMIV230-01 (5 µg/ml) were loaded onto sensors until a response of 0.5 nm, followed by another 60 s baseline step. Association measurements using nanobodies were performed using a two-fold dilution series of untagged Pfs230 D1D2 from 6 to 200 nM or untagged Pfs230 D1 from 0.8 to 100 nM for 180 s, and dissociation was measured in kinetics buffer for 180 s. For Fc-tagged nanobodies and LMIV230-01 association measurements were performed using two-fold dilution series of untagged Pfs230 D1D2 or untagged Pfs230 Pro-D1D2 from either 3 to 100 nM or 94–3000 nM for 180 s. Dissociation was measured in kinetics buffer for 180 s. Sensor tips were regenerated using five cycles of 5 s in 300 mM imidazole pH 7.5 and 5 s in kinetics buffer for NTA-sensors, or five cycles of 5 s in 100 mM glycine pH 1.5 and 5 s in kinetics buffer for AHC-sensors. Baseline drift was corrected by subtracting the response of a nanobody-loaded sensor incubated in kinetics buffer only. Curve fitting analysis was performed with Octet Data Analysis 10.0 software using a global fit 1 : 1 model to determine *K*_D_ values and kinetic parameters. Curves that could not be fitted well were excluded from the analyses. The mean kinetic constants reported are the result of two to three independent experiments.

For epitope binning experiments, 30 nM untagged Pfs230 D1D2 was pre-incubated with each nanobody at a 10-fold molar excess for 1 h at RT. A 30 s baseline step was established between each step of the assay. The NTA sensors were first loaded with 10 µg/ml of nanobody for 5 min. The sensor surface was then quenched by dipping into 20 µg/ml of an irrelevant nanobody for 5 min. Nanobody-loaded sensors were then dipped into premixed solutions of Pfs230 D1D2 and nanobody for 5 min. Nanobody-loaded sensors were also dipped into Pfs230 D1D2 alone to determine the level of Pfs230 D1D2 binding to immobilized nanobody in the absence of other nanobodies. Percentage competition was calculated by dividing the max response of the premixed Pfs230 D1D2 and nanobody solution binding by the max response of Pfs230 binding alone, multiplied by 100.

### *P. falciparum* maintenance, gametocyte culture and mosquito infection

*P. falciparum* NF54 asexual parasites were cultured in human type O-positive erythrocytes (Melbourne Red Cross) at 4% haematocrit in RPMI HEPES supplemented with 50 µg/ml hypoxanthine, 0.2% (w/v) NaHCO_3,_ and 10 mM D-Glucose) containing 5% heat-inactivated human serum and 5% Albumax (ThermoFisher Scientific).

Gametocytes for transmission to mosquitoes were generated using the ‘crash' method [38]. Briefly, gametocyte cultures were set up with 0.65% synchronized ring stage parasite in 4% hematocrit. Gametocyte cultures were maintained in RPMI 1640 medium supplemented with 25.96 mM HEPES, 50 µg/ml hypoxanthine, 0.2% (w/v) NaHCO_3,_ and 10 mM D-Glucose) containing 10% heat-inactivated human serum and with daily media changes.

Three- to five-day old female *Anopheles stephensi* mosquitoes were fed on asynchronous gametocytes diluted to 0.2% stage V gametocytemia, via a water-jacketed glass membrane feeder. Three- to four hours post-blood feeding mosquitoes were knocked down with CO_2_ to sort fully engorged from unfed mosquitoes. Engorged mosquitoes were transferred to tents and were provided sugar cubes and water wicks ad libitum. Seven days post-blood feeding, midguts were dissected from cold-anesthetized and ethanol sacrificed mosquitos, stained with 0.1% mercurochrome, and the oocyst numbers were enumerated.

### Western blotting

For western blotting with recombinant protein, 250 ng untagged Pfs230 D1D2 was separated on a 4–12% Bis-Tris gel in non-reducing and reducing conditions and transferred onto a PVDF membrane using iBlot 2 (Invitrogen). The membrane was blocked with 10% milk in PBST for 1 h at RT and subsequently probed with either 0.5 µg/ml anti-Pfs230 D1D2 nanobodies, 0.5 µg/ml WNb7 nanobody, or the pre- and post-immune alpaca serum at 1/500 dilution overnight at 4°C. Following nanobody incubation, the membrane was washed for 5 minutes three times with 1x PBST and probed with 1/5000 polyclonal rabbit anti-nanobodies (WEHI), followed by 1/5000 anti-rabbit HRP, both for 1 h at RT. All primary nanobodies, secondary, and tertiary antibodies were prepared in 1% milk in 1× PBST. Chemiluminescent detection was performed using SuperSignal™ West Pico PLUS Chemiluminescent Substrate (Pierce) and imaging was done with a Chemidoc imager (Bio-Rad). To demonstrate equivalent loading of the recombinant proteins, 500 ng of reduced and non-reduced Pfs230 D1D2 were stained on SDS–PAGE using Instant Blue (Invitrogen).

For western blotting of gametocytes, synchronized stage V gametocyte culture was prepared as described before [39]. The parasite material (5 µl) was loaded on a 3–8% Tris-Acetate gel for 75 min at 150 V under reducing and non-reducing conditions. Proteins were transferred onto a nitrocellulose membrane, and subsequently probed with primary nanobodies as described above. Human monoclonal anti-Pfs230 LMIV230-01 was used as a positive control at 0.5 µg/ml. For detection using nanobodies, the blot was incubated with 1/500 HRP-conjugated goat anti-llama IgG (Sapphire Biosciences) whilst detection with LMIV230-01 was with 1/5000 HRP-conjugated anti-human antibody. Nanobody/antibody preparation, washing and visualization of the blot was carried out as described above.

### Immunofluoresence assays

Stage V gametocytes were harvested from culture and thin blood smears were made on glass slides and fixed in equal parts of ice-cold acetone and methanol for 20 min and allowed to air dry before use. The slides were washed three times in 1× PBS prior to addition of the primary antibodies. The primary antibodies were diluted in 3% BSA/PBS. Anti-Pfs230 LMIV-230-01 was used at a final concentration of 7.5 µg/ml and the Pfs230 nanobodies F5, F10 and the negative control SARS-CoV-2 nanobody WNb7 were used at 4 µg/ml [24]. Primary antibodies were incubated on the slide for 2 h. After washing, goat anti-llama IgG conjugated to Alexa 488 and goat anti-human IgG conjugated to Alexa 647 (both 1:1000 in 3% BSA/PBS) were used as secondary antibodies and incubated for 1 h. DAPI at 2 µg/ml was added for 10 min, and the slide was washed as previously described. The slides were mounted in 90% glycerol containing 0.2% p-phenylenediamine and a coverslip was added and sealed with nail polish. Slides were imaged on a DeltaVision Elite Restorative Widefield Deconvolution System (GE Healthcare) and a 100x UPLS Apo (1.4NA) objective lens under oil immersion. Samples were excited with solid state illumination (Insight SSI, Lumencor). The filter sets for DAPI (excitation wavelength 390/18, emission wavelength 435/48); FITC (Ex475/28, Em523/26); Cy5 Ex 632/22, 676/34 nm. Images were deconvolved using the default settings in SoftWoRx 5.0 (GE Healthcare) acquisition software. Images were further processed using FIJI ImageJ software and the maximum projection images used in the figures (version 2.3.0/1.563r).

### Exflagellation center formation assay

The exflagellation assay was performed as described previously [40]. Briefly, 15 µl of resuspended gametocyte culture was transferred to a 37°C pre-warmed microcentrifuge tube. An amount of 15 µl of ookinete media (RPMI 1640 medium supplemented with 26 mM HEPES, 50 µg/ml hypoxanthine, 0.2% (w/v) NaHCO_3_, 10% heat-inactivated human serum, and 100 µM xanthurenic acid) with nanobodies was added to the resuspended culture to induce gamete formation. After 15 min incubation at RT, parasites were transferred to a Neubauer chamber and the exflagellation was observed by phase contrast microscopy with a 40× objective. The number of exflagellation center and red blood cells (RBCs) were counted and recorded from the four 4 × 4 outer grids and 16 small square of the central grid, respectively. The percentage of exflagellation centers can be calculated from the number of exflagellation centers divided by the number of RBCs.

### Size exclusion chromatography analyses of nanobody-Pfs230 complexes

Protein-protein complexation was carried out by incubating Pfs230 Pro-D1D2 or Pfs230 D1D2 with nanobodies or LMIV230-01 at different molar ratios for 1 h on ice. Samples were applied onto a SD200 3.2/300 column pre-equilibrated in 20 mM HEPES pH 7.5, 150 mM NaCl. Equivalent amounts of single proteins were run for comparison of retention volumes to assess complex formation.

## Data Availability

Coordinates and structure factors have been deposited in the Protein Data Bank (PDB) under PDB ID 7USR [41] for Pfs230 D1D2, 7USS [42] for Pfs230 Pro-D1D2, 7UST [43] for Pfs230 D1-F5 and 7USV [44] for Pfs230 D1-F10.

## Open Access

Open access for this article was enabled by the participation of University of Melbourne in an all-inclusive *Read & Publish* agreement with Portland Press and the Biochemical Society under a transformative agreement with CAUL.

## Abbreviations

BLI: bio-layer interferometry
CDR: complementary-determining regions
GPI: glycosylphosphatidylinositol
RBCs: red blood cells
TEV: Tobacco Etch Virus
TRA: transmission reducing activity
