# Supplementary material for: Nanobodies against Pfs230 block Plasmodium falciparum transmission
Source: Biochem J. 2022 Dec 22;479(24):2529–46. doi: 10.1042/BCJ20220554 (PMC9788556; doi:10.1042/BCJ20220554)
Supplement: Supplementary Material [file BCJ-479-2529-s1.pdf]

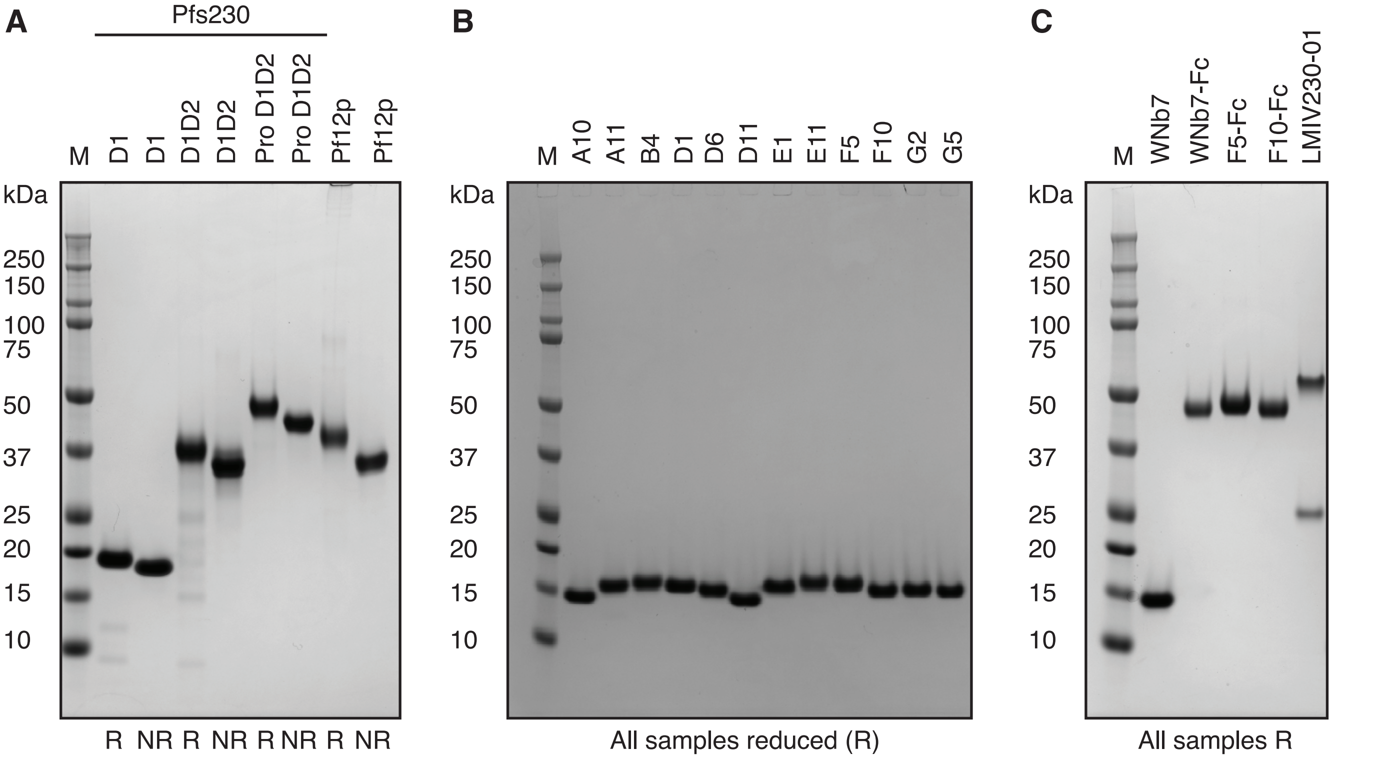

Fig. S1. Coomassie-stained SDS-PAGE gels of purified recombinant proteins. (A) Recombinant Pfs230 proteins and Pf12p under reducing (R) as well as non-reducing (NR) conditions. In each case 2 µg of protein was loaded. Moleular weight marker with corresponding sizes are indicated (M). (B) Anti-Pfs230 nanobodies under reducing condiditons. (C) Control nanobody WNb7, Fc-tagged nanobodies and mAb LMIV230-01 under reducing conditions.

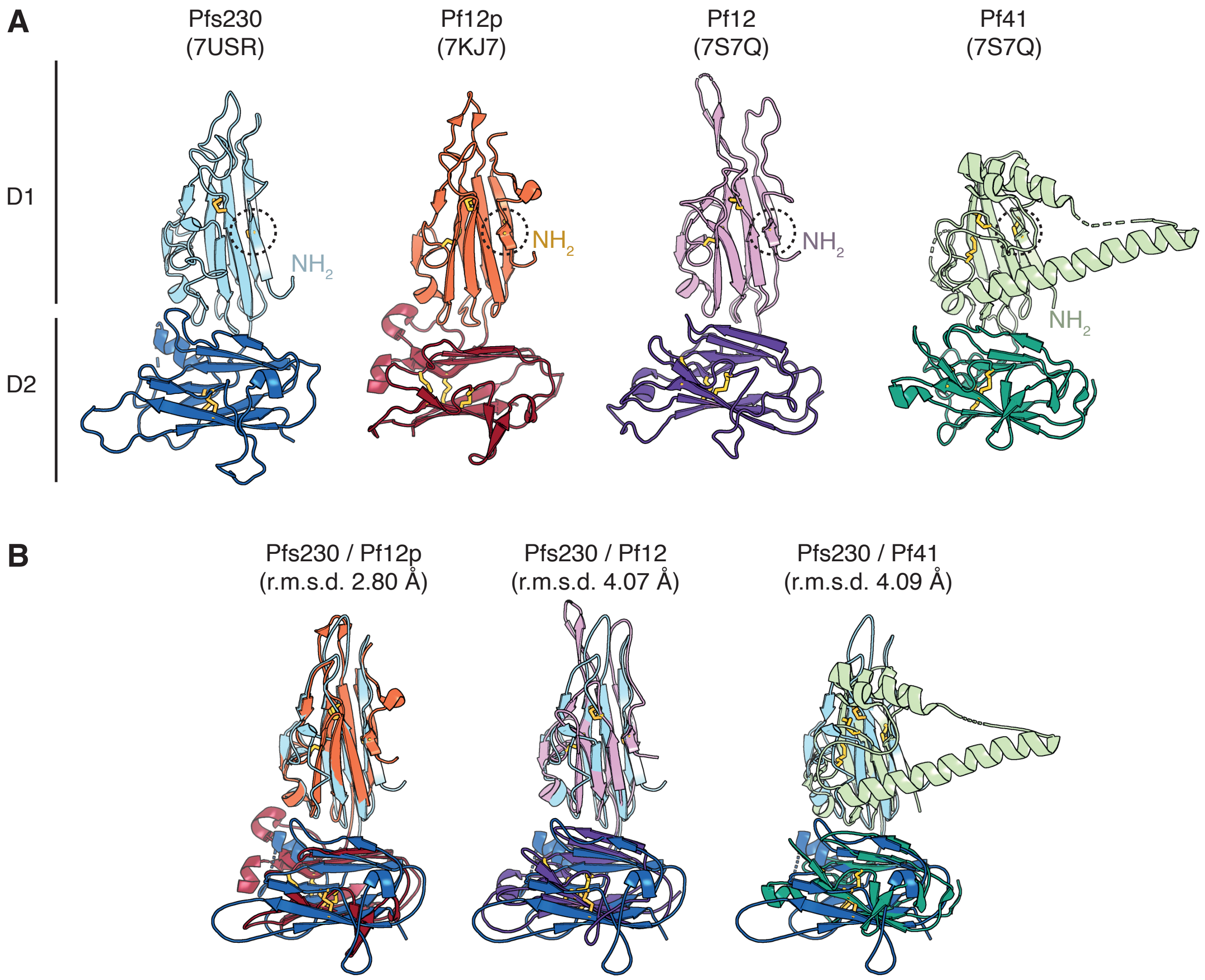

Fig. S2. Crystal structures of 6-cys protein tandem pairs. (A) Crystal structures of different 6-cys protein family members are shown; Pfs230 (blue), Pf12p (orange), Pf12 (purple) and Pf41 (green) with A-type 6-cys domains (D1) shown in light shade and B-type 6-cys domains (D2) shown in darker shade. Disulfide bonds are shown in ball and stick representation (yellow). Dashed circle indicates disulfide bonds that connect the first two β-strands in A-type domains. Corresponding PDB ID are indicated. (B) Structural alignment of Pfs230 D1D2 with Pf12p (PDB ID 7K7J), Pf12 (PDB ID 7S7Q) and Pf41 (PDB ID 7S7Q).

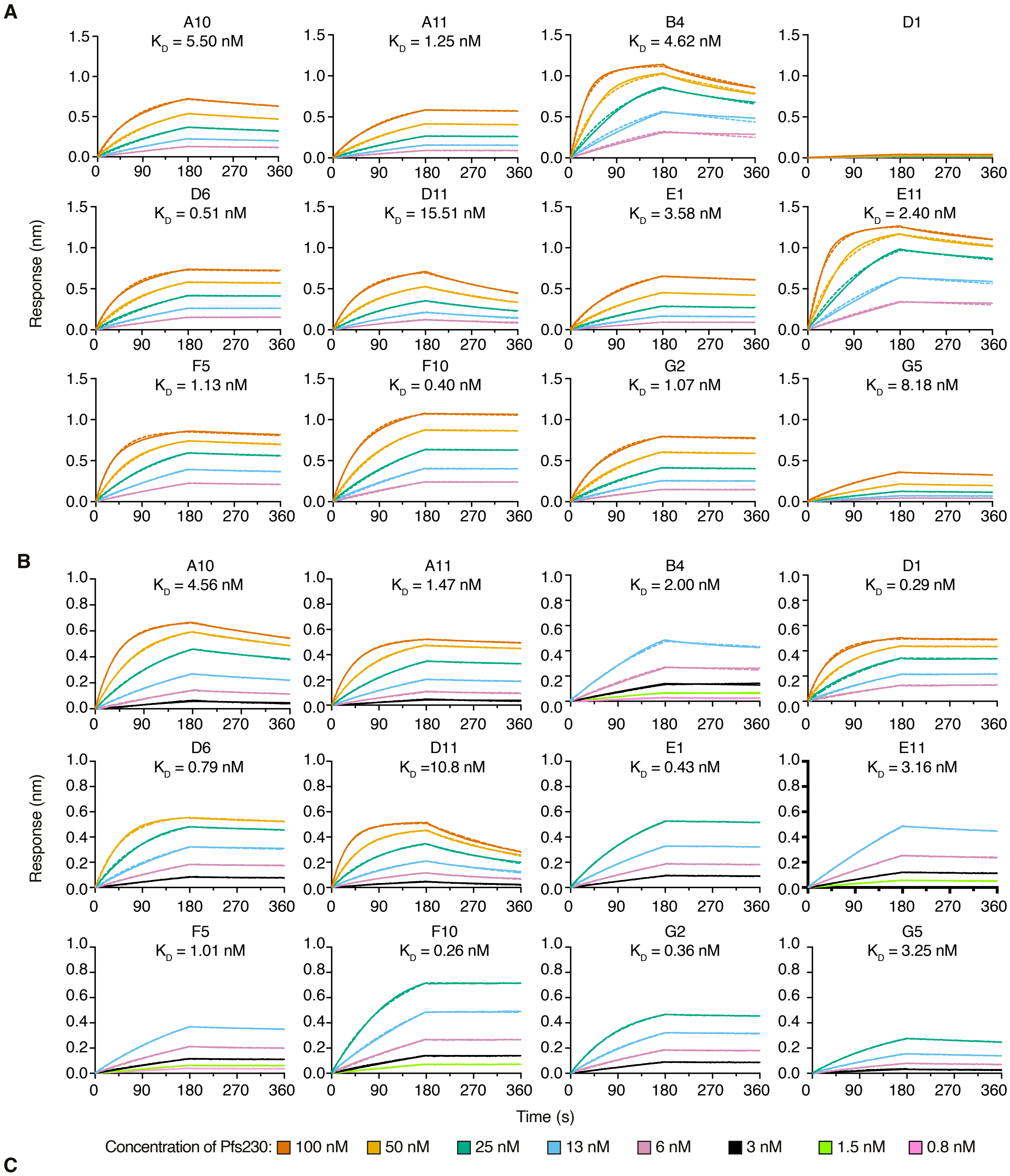

C

	Pfs230 D1D2			Pfs230 D1		
Pfs230 Nbs	K_D (nM)	k_a (x 10^5 $M^{-1}s^{-1}$)	k_d (x 10^{-5} s^{-1})	K_D (nM)	k_a (x 10^5 $M^{-1}s^{-1}$)	k_d (x 10^{-5} s^{-1})
A10	5.87 (± 0.52)	1.37 (± 0.04)	80.50 (± 9.76)	3.18 (± 1.95)	2.53 (± 0.17)	78.80 (± 44.12)
A11	1.43 (± 0.25)	1.07 (± 0.02)	15.20 (± 2.97)	1.56 (± 0.13)	2.49 (± 0.33)	39.00 (± 8.20)
B4	4.80 (± 0.25)	3.17 (± 0.08)	152.00 (± 11.31)	1.71 (± 1.62)	14.05 (± 12.52)	138.50 (± 13.44)
D1	N/A	N/A	N/A	0.14 (± 0.20)	2.35 (± 0.35)	3.72 (± 5.24)
D6	0.63 (± 0.17)	1.73 (± 0.06)	10.84 (± 2.50)	0.39 (± 0.56)	3.61 (± 0.78)	15.76 (± 22.27)
D11	16.15 (± 0.92)	1.62 (± 0.00)	261.50 (± 14.85)	8.31 (± 3.53)	3.09 (± 0.06)	256.00 (± 104.70)
E1	3.91 (± 0.46)	1.05 (± 0.03)	40.95 (± 3.89)	0.75 (± 0.29)	2.52 (± 0.27)	19.00 (± 8.66)
E11	2.54 (± 0.19)	2.90 (± 0.04)	73.35 (± 4.31)	2.61 (± 0.90)	2.36 (± 1.26)	53.93 (± 6.96)
F5	1.22 (± 0.12)	2.58 (± 0.08)	31.30 (± 2.12)	0.81 (± 0.20)	4.07 (± 1.15)	31.57 (± 2.49)
F10	0.45 (± 0.08)	1.81 (± 0.04)	8.19 (± 1.28)	0.12 (± 0.13)	3.89 (± 0.52)	4.80 (± 5.34)
G2	1.11 (± 0.06)	1.44 (± 0.04)	15.95 (± 0.21)	0.49 (± 0.35)	3.72 (± 0.27)	17.93 (± 12.45)
G5	9.09 (± 1.29)	0.63 (± 0.03)	57.90 (± 10.75)	3.36 (± 1.70)	1.94 (± 0.14)	63.53 (± 27.90)

Fig. S3. Nanobody affinities to Pfs230. (A) Representative binding curves of different Pfs230 D1D2 concentrations to immobilized nanobodies are shown and were fitted using a 1:1 binding model. (B) Representative binding curves of different Pfs230 D1 concentrations to immobilized nanobodies are shown and were fitted using a 1:1 binding model. Corresponding KD values are indicated. (B) Table containing determined kinetic and affinity data from two or three independent experiments showing the mean and the standard deviation (SD).

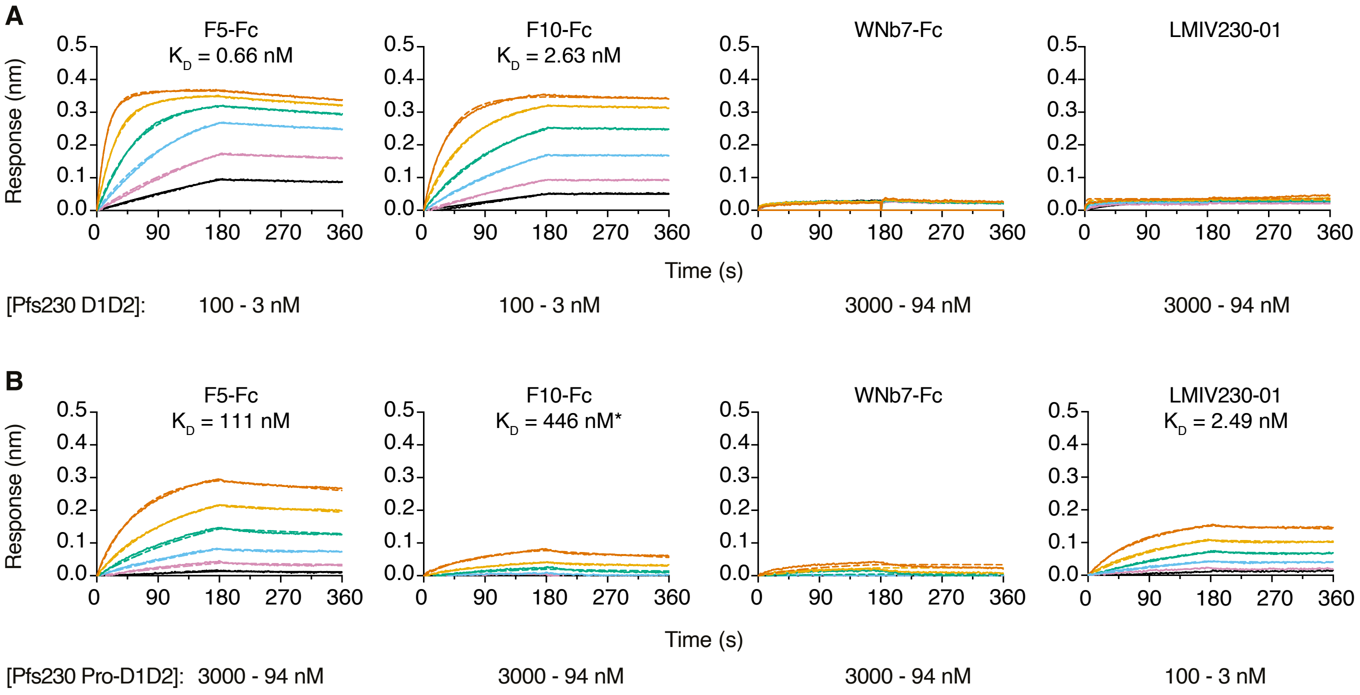

C

	Pfs230 D1D2			Pfs230 Pro-D1D2		
Pfs230 Nbs	KD (nM)	k_a (x 10^3 $M^{-1}s^{-1}$)	k_d (x 10^{-5} s^{-1})	KD (nM)	k_a (x 10^3 $M^{-1}s^{-1}$)	k_d (x 10^{-5} s^{-1})
F5-Fc	0.57 (± 0.09)	607.50 (± 54.50)	35.15 (± 8.35)	117.50 (± 6.50)	5.49 (± 0.01)	64.45 (± 3.45)
F10-Fc	0.13 (± 0.13)	314.00 (± 11.00)	3.99 (± 3.98)	632.50 (± 186.50)*	2.99 (± 0.98)*	171.00 (± 6.00)*
WNb7-Fc	N/A	N/A	N/A	N/A	N/A	N/A
LMIV230-01	N/A	N/A	N/A	3.98 (± 1.49)	116.15 (± 20.85)	43.05 (± 9.05)

Fig. S4. Fc-tagged nanobody affinities to Pfs230. (A) Representative binding curves of different Pfs230 D1D2 concentrations to immobilized Fc-tagged nanobodies or LMIV230-01 are shown and were fitted using a 1:1 binding model. (B) Representative binding curves of different Pfs230 Pro-D1D2 concentrations to immobilized Fc-tagged nanobodies or LMIV230-01 are shown and were fitted using a 1:1 binding model. Notably, for F10-Fc binding to Pfs230 Pro-D1D2 (*) only three binding curves were used for fitting. All other affinities and kinetics were determined using five or six binding curves respectively. Corresponding KD values are indicated. (C) Table containing determined kinetic and affinity data from two independent experiments showing the mean and the standard error of mean.

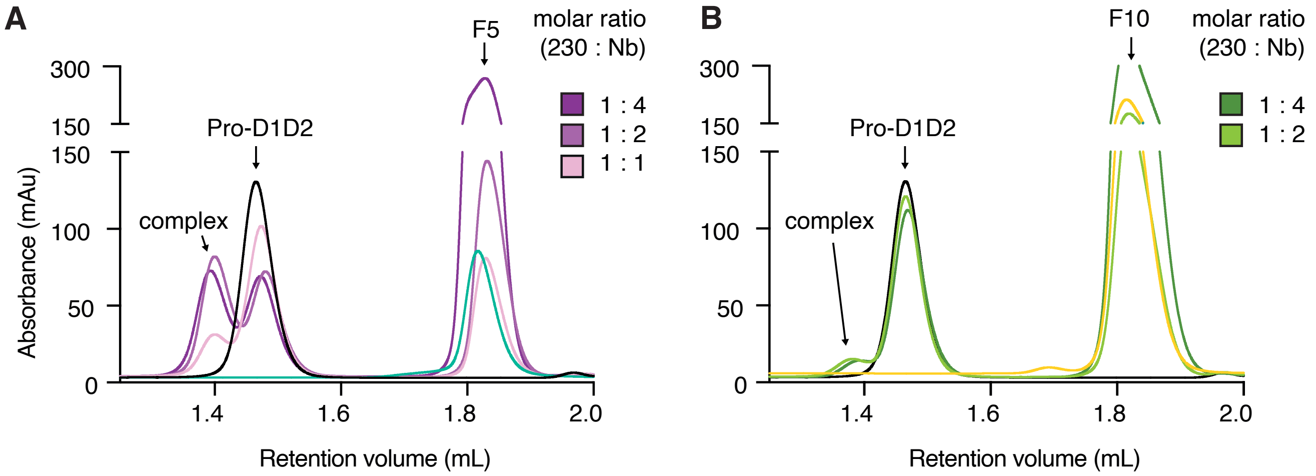

Fig. S5. Size shift assay of Pfs230 Pro-D1D2 with nanobody F5 and F10 respectively. (A) Size exclusion chromatography (SEC) runs of Pfs230 Pro-D1D2 mixed with F5 in different molar ratios (1:1, 1:2 and 1:4 from light to dark purple). SEC runs of Pfs230 Pro-D1D2 (black) and F5 (green) alone are shown in addition. A 1:1 molar mix of F5 with Pfs230 Pro-D1D2 showed a partial shift of the recombinant proteins to higher molecular weight corresponding to a 1:1 complex of nanobody and Pfs230 Pro-D1D2. More complex was formed with a 2:1 molar ratio of nanobody to Pfs230 but this trend did not further increase with a 4-fold molar excess of nanobody. (B) SEC runs of Pfs230 Pro-D1D2 mixed with F10 in different molar ratios (1:2 and 1:4 from light and dark green). SEC runs of Pfs230 Pro-D1D2 (black) and F10 (orange) alone are shown in addition. A small amount of F10-Pfs230 Pro-D1D2 complex was formed using 2, or 4 molar excess of F10.

Supplementary Table S1. Amino acid sequences of anti-Pfs230 nanobodies

**Nb**	**full VHH**
A10	QVQLQESGGGLVQAGSSLRLSCAASGGTFSNYAMGWFRQAPGKEREFVAAIGWSGGTYYTDSVEGRFTISRD NAKNTVYLQMNSLKPEDTAVYYCAAKRSGSSWSERGYDYWGQGTQVTVSS
A11	QVQLQESGGGLVQPGESLRLSCAASGRTSSSYAMGWFRQAPGKEREFVASISWSGGSTYYADSVKGRFTLSRD NAKNTVYLQMNSLKPEDTAVYYCAADGIFYSDYPLSVDDFHSWGQGTQVTVSS
B4	QVQLQESGGGLVQPGGSLRLSCAASGFTLDYYGIGWFRQAPGKEREGVSCISSSDGSTYYADSVKGRFTISRDN AKNTVYLQMNSLKPEDTAVYYCAADLTGIGCTTAQAMGVIGDFGDFGSWGQGTQVTVSS
D1	QVQLQESGGGLVQAGDSLRLSCTASGRTFSNHAMGWFRQAPGKEREIVAVIFGTGRNSWYVDSVKGRFTISRD NAKNTVALQMNRLKPEDTAVYYCASGRTWYSGSSMAEYDYWGQGTQVTVSS
D6	QVQLQESGGGLVQAGDSLRLSCAASGRTFSSYFMGWFRQAPGKEREFVAAISWSGGSTYYADSVKGRFTISRD NVKNTVYLQMNSLEPEDAAVYYCAGGGSYYPMSPRNGMDYWGKGTQVTVSS
D11	QVQLQESGGGLVQAGSSLRLSCAASGRTFSSYAMGWFRQAPGKEREFVAAIGWSGGTYYTDSVEGRFTISRDN AENTVYLQMNSLKPEDTAVYYCAAKASGSSWSERGYDYWGQGTQVTVSS
E1	QVQLQESGGGLVQPGGSLRLSCAASGRTFSSYFMGWFRQAPGKGRVFVASISWSGGSTYYADSVKGRFTISRD NAENTVYLQMNSLKPEDTAVYYCAAGTRATPLDWASGMDYWGKGTQVTVSS
E11	QVQLQESGGGLVQPGGSLRLSCAASGFTSDYYGIGWFRQAPGKEREGVACISSSDGSTYYADSVKGRSTISRDN AKNTVYLQMNSLKPEDTAVYYCAADLSGIGCTTAQAMDVIGDFGDFGSWGQGTQVTVSS
F5	QVQLQESGGGLVQPGGSLRLSCAASGFTLDRYAIGWFRQAPGKEREGVSCISSSDGSTYYADSVKGRFTISRDN AKNTVYLQMNSLKPEDTAVYYCARDHGPCTVLADILYDYGMDYWGKGTQVTVSS
F10	QVQLQESGGGLVQAGGSLRLSCAASGRTFSDYFMGWFRQAPGKEREFVAAVSWSGGSTYYADSVKGRFTISR DNAKNTVFLQMNSLKPEDTAVYYCAGGGSYYPMSPYDGMDYWGKGTQVTVSS
G5	QVQLQESGGGLVQAGGSLRLSCAASGRTFNDYAMYWFRQAPGKEREFVADITWSGSSTYYADSVKGRFTISR DNAKNTVFLQMNSLKPEDTALYYCAADSRYYGIGIGTPRYWGQGTQVTVSS
G2	QVQLQESGGGLVQAGGSLRLSCAASGRTFSNYFMGWFRQAPGKEREFVAAVSWSGGSTYYADSVKGRLTISR DNAKNTVYLQMNSLKPEDTAVYYCAAGERYFPMEPRRGYDYWGQGTQVTVSS

Supplementary Table S2. Raw data of the standard membrane feeding assay

Group	Experiment 1		Experiment 2		Experiment 3	
	**Mean oocysts/midgut (min-max)**	**% infected mosquitoes**	**Mean oocysts/midgut (min-max)**	**% infected mosquitoes**	**Mean oocysts/midgut (min-max)**	**% infected mosquitoes**
Untreated	28.1 (2-65)	76.7	31.6 (4-68)	80.0	27.1 (1-67)	93.3
PBS	21.0 (4-66)	70.0	28.7 (8-85)	66.7	20.4 (4-86)	73.3
WNb7-Fc	24.3 (3-61)	76.7	35.6 (19-87)	76.7	24.6 (3-51)	70.0
LMIV230-01	0.8 (1-10)	23.3	1.7 (1-11)	36.7	0.5 (1-7)	23.3
F5-Fc	6.0 (1-22)	73.3	4.1 (1-30)	70.0	3.4 (2-18)	53.3
F10-Fc	5.9 (1-38)	60.0	6.9 (1-32)	56.7	3.7 (1-20)	46.7
F5-Fc + F10-Fc	4.6 (1-21)	76.6	3.8 (1-25)	80.0	4.1 (1-14)	73.3

Supplementary Table S3. Interactions Pfs230 D1 – F5 complex

Pfs230	Group	F5	Location	Group	Distance
Hydrogen bonds					
Lys 653	NZ	Ala 107	CDR3	O	3.0
Leu 658	O	Tyr 113	CDR3	OH	3.0
Glu 660	N	Tyr 113	CDR3	OH	3.6
Arg 720	NH1	Pro 102	CDR3	O	3.2
Arg 720	NH1	Thr 104	CDR3	OG1	2.9
Salt bridges					
Lys 659	NZ	Glu 44	FR2	OE2	3.2
Arg 720	NH2	Asp 99	CDR3	OD2	2.8
Other interfacing residues in Pfs230					
Pro 606	Lys 607	Val 608	Lys 610	Val 642	Leu 651
Thr 656	Lys 657	Tyr 703	Ile 705	Asp 707	Ser 709
Lys 710	Ile 722				
Other interfacing residues in F5					
Arg 31	Arg 45	Glu 46	Gly 47	Ser 53	His 100
Gly 101	Cys 103	Leu 106	Asp 108	Ile 109	Leu 110
Tyr 111	Gly 114	Asp 116			

Supplementary Table S4. Interactions Pfs230 D1 – F10 complex

Pfs230	Group	F10	Location	Group	Distance
		Hydrogen bonds			
Glu 590	OE1	Ser 101	CDR3	OG	2.9
Glu 590	O	Tyr 102	CDR3	N	3.0
Glu 590	N	Gly 100	CDR3	O	3.7
Val 592	N	Tyr 102	CDR3	O	2.9
Glu 612	O	Tyr 59	CDR2	OH	2.7
Lys 614	NZ	Thr 58	CDR2	O	2.8
Lys 614	N	Tyr 59	CDR2	OH	3.0
Lys 614	O	Ser 57	CDR2	OG	3.5
Asn 616	ND2	Gly 56	CDR2	O	2.8
Asn 616	OD1	Ser 57	CDR2	N	3.8
Asn 616	OD1	Ser 57	CDR2	OG	3.0
Glu 617	OE2	Ser 52	CDR2	OG	2.6
Glu 617	OE1	Ser 54	CDR2	N	2.9
		Salt bridges			
Lys 589	NZ	Asp 31	CDR1	OD1	3.9
		Other interfacing residues in Pfs230			
Thr 587	Asn 588	Tyr 591	Cys 593	Asp 594	Phe 595
Lys 609	Lys 610	Cys 611	Val 613	Val 615	Ile 620
Ile 625					
		Other interfacing residues in F10			
Ser 30	Phe 33	Trp 53	Tyr 103	Pro 104	Met 105
Ser 106					
